# Utilization of Neoadjuvant Chemotherapy Varies in the Treatment of Women with Invasive Breast Cancer

**DOI:** 10.1371/journal.pone.0084535

**Published:** 2013-12-20

**Authors:** Adedayo A. Onitilo, Jill K. Onesti, Richard M. Single, Jessica M. Engel, Ted A. James, Erin J. Aiello Bowles, Heather Spencer Feigelson, Tom Barney, Laurence E. McCahill

**Affiliations:** 1 Department of Hematology and Oncology, Marshfield Clinic, Weston, Wisconsin, USA and Clinical Epidemiology Unit, School of Population Health, University of Queensland, Brisbane, Australia; 2 Grand Rapids Medical Education Partners, Grand Rapids, Michigan, USA; 3 University of Vermont, Burlington, Vermont, United States of America; 4 Marshfield Clinic Cancer Care at St Michaels Hospital, Stevens Point, Wisconsin, United States of America; 5 University of Vermont College of Medicine, Burlington, Vermont, United States of America; 6 Group Health Research Institute, Seattle, Washington, United States of America; 7 Kaiser Permanente Colorado, Denver, Colorado, United States of America; 8 Van Andel Research Institute, Grand Rapids, Michigan, United States of America; Davidoff Center, Israel

## Abstract

**Background:**

Treatment with neoadjuvant chemotherapy (NAC) has made it possible for some women to be successfully treated with breast conservation therapy (BCT ) who were initially considered ineligible. Factors related to current practice patterns of NAC use are important to understand particularly as the surgical treatment of invasive breast cancer has changed. The goal of this study was to determine variations in neoadjuvant chemotherapy use in a large multi-center national database of patients with breast cancer.

**Methods:**

We evaluated NAC use in patients with initially operable invasive breast cancer and potential impact on breast conservation rates. Records of 2871 women ages 18-years and older diagnosed with 2907 invasive breast cancers from January 2003 to December 2008 at four institutions across the United States were examined using the Breast Cancer Surgical Outcomes (BRCASO) database. Main outcome measures included NAC use and association with pre-operatively identified clinical factors, surgical approach (partial mastectomy [PM] or total mastectomy [TM]), and BCT failure (initial PM followed by subsequent TM).

**Results:**

Overall, NAC utilization was 3.8%l. Factors associated with NAC use included younger age, pre-operatively known positive nodal status, and increasing clinical tumor size. NAC use and BCT failure rates increased with clinical tumor size, and there was significant variation in NAC use across institutions. Initial TM frequency approached initial PM frequency for tumors >30-40mm; BCT failure rate was 22.7% for tumors >40mm. Only 2.7% of patients undergoing initial PM and 7.2% undergoing initial TM received NAC.

**Conclusions:**

NAC use in this study was infrequent and varied among institutions. Infrequent NAC use in patients suggests that NAC may be underutilized in eligible patients desiring breast conservation.

## Introduction

The efficacy of neoadjuvant chemotherapy (NAC) in achieving successful breast conservation for women initially considered ineligible for breast conservation therapy (BCT) was demonstrated more than 15 years ago [1]. Current practice patterns of NAC use, however, are not well-described, particularly as the surgical treatment of invasive breast cancer has changed in the past 20 years. Partial mastectomy (PM) has become more common as the initial surgical procedure for management of the primary breast tumor [2,3]. This shift is largely due to the results of several randomized controlled trials and meta-analyses that established BCT to be as effective as total mastectomy (TM) with regard to overall patient survival [4,5] and the 1990 National Institutes of Health Consensus Conference [6] recommendation of BCT as an alternative to TM. More recently, TM rates appear to be increasing secondary to a variety of proposed factors, although this remains controversial [3,7-9]. Clinical conditions or patient-related factors associated with greater utilization of initial mastectomy include larger tumor size, multicentric breast cancer, family history of breast cancer, race, younger age, pre-operative breast magnetic resonance imaging (MRI) utilization, lower socioeconomic status, greater living distance from a radiation facility, patient preference, and provider preference [10-14].

Commonly accepted indications for NAC include the treatment of patients presenting with inflammatory breast cancer or T4 lesions (direct invasion of tumor into chest wall or skin) as well as treatment of women with initially operable tumors (T1c-3, N0-2, M0) who desire BCT but would be considered ineligible based on tumor size or tumor to breast size ratio [15,16]. A significant amount of clinical research has demonstrated the efficacy and safety of NAC in allowing patients initially considered ineligible for BCT to successfully undergo BCT without compromising patient overall survival. The National Surgical Adjuvant Breast and Bowel Project-18 (NSABP B-18) trial found NAC to be equally efficacious to post-operative chemotherapy in overall survival rates, while demonstrating a moderate increase in the percentage of women who were candidates for BCT, as did NSABP B-27 [1,17]. Similarly, investigators at the Milan Cancer Institute reported even greater success with NAC use, allowing for BCT in >50% of patients with large tumors (>5.0 cm) who were initially considered ineligible for BCT [18]. The results of these and other trials have led to endorsement of NAC by the National Comprehensive Cancer Network (NCCN) and an international panel of experts [19,20].

Under-utilization of effective treatments and other forms of unexplained clinical variation are recognized barriers to efficient and optimal health care. Studies have demonstrated wide variation in both regional and provider-level practice patterns that can result in decreased quality of care due to underuse, overuse, or misuse of health care services [21,22]. Comparison of NAC utilization between different health care organizations outside of clinical trials has not been well reported; therefore, with our study, we sought to examine the current pattern of NAC utilization among four geographically diverse healthcare institutions and identify factors associated with NAC use, particularly in the scenario of failed breast conservation. 

## Methods

### Ethics Statement

Approval to conduct the study, with waiver of consent to collect patient and provider-level data, was obtained from the Institutional Review Board (IRB) of Fletcher Allen Health Care/University of Vermont, the Kaiser Permanente Colorado IRB, and the Group Health Cooperative IRB. The Marshfield Clinic Research Foundation’s IRB ceded approval to Kaiser Permanente Colorado’s IRB. Criteria for waiver of consent included minimal risk study (retrospective review of data already in existence) and only a de-identified data set that was shared and used for analysis with the Van Andel Research Institute, where the database is housed. 

The source of data for this study is the Breast Cancer Surgical Outcomes (BRCASO) database. The BRCASO study was an American Recovery and Reinvestment Act (ARRA) funded comparative effectiveness study intended to evaluate and compare initial surgical treatment and outcomes of patients with newly diagnosed breast cancer from January 2003 to December 2008 [23]. The BRCASO database is a multi-institutional database of female patients (age >18 years) who underwent initial surgical treatment for breast cancer at four institutions including Fletcher Allen Health Care (affiliated with the University of Vermont) and three sites of the Cancer Research Network (CRN): Group Health Cooperative (Washington State), Kaiser Permanente of Colorado, and the Marshfield Clinic (Wisconsin). BRCASO study general eligibility criteria included female gender, pathologic confirmation of stage 0–IV invasive breast cancer, and initial surgical treatment (ie, PM or TM) performed by a study institution-employed surgeon. Women with conditions commonly associated with ineligibility for BCT including multifocal breast cancer, inflammatory breast cancer, and prior history of ipsilateral breast cancer or radiation therapy were excluded from analyses. We also excluded women with a pre-operative diagnosis of ductal carcinoma in situ (DCIS). Inclusion and exclusion criteria were designed to identify patients who would be candidates for BCT if tumor size prior to initial surgery permitted. Available data include that pertaining to surgical procedures, surgeon information, cancer information, whether or not neoadjuvant chemotherapy was given, and patient demographics and characteristics. 

Each of the four study sites contributed their data to the BRCASO database. Data were collected both electronically and by extensive manual review of the medical records [23]. The three CRN affiliated study sites utilized electronically captured data elements from the Virtual Data Warehouse (VDW), a standardized source of data housed at each CRN site with standard formatting across sites. Data incorporated into the VDW includes patient information related to clinical care and health plan enrollment. One programmer familiar with the VDW wrote the programs for identifying patients and for collection of available and relevant data at the CRN sites, and the BRCASO database was prefilled with these data. Experienced chart abstractors at all sites underwent group training for this study in order to collect and report data in a standard fashion at each site. A detailed manual of instructions and definitions was available for abstractor use. Data that were specifically reviewed manually included surgical reports and pathology reports. Abstractors at each site reviewed charts from their own site and entered data directly into a database that was saved in the BRCASO database at the Van Andel Research Institute.

For this study evaluating NAC, we specifically evaluated the utilization of pre-operative systemic chemotherapy as it related to the clinician’s best estimate of tumor size before treatment initiation. The largest tumor diameter reported by radiology studies was used for analysis, and when not available, the tumor size reported by a clinician’s physical exam was used. All tumor size estimations used for analysis were obtained before chemotherapy and initial surgery. Likewise, preoperative tumor markers were analyzed, including estrogen and progesterone receptor status. Her2Neu receptor status was not analyzed, as it was not consistently available before surgery based on biopsy results alone.

NAC was selected according to the treating oncologist of each institution; specific treatment regimens were not analyzed. Patients receiving neoadjuvant endocrine therapy alone were not included in the analysis. We did not analyze the conversion rate for patients initially ineligible for BCT who then successfully underwent BCT after NAC, given the retrospective limitations. For study purposes, BCT failure was defined as patients undergoing TM after an initial PM for the same diagnostic event. PM requiring re-excision was not considered BCT failure if the final procedure was a PM.

Bivariate analyses relating demographic and clinical variables with NAC use were performed using Pearson’s Chi-square test. A Markov chain montecarlo (MCMC) approximation to Fisher’s exact test was used when cell counts were too small for the Chi-square test. Variables that were significant in the univariate analyses were included in a multivariable logistic regression model. P-values are reported for 2-sided tests and are not adjusted for multiple comparisons. All data analyses were performed using SAS version 9.2 (SAS Institute, Cary, NC).

## Results

A total of 4527 patient records were reviewed in the BRCASO database and consisted of 4630 diagnostic events (including additional tumors in the contralateral breast). Of these patients, 2871 women with 2907 diagnostic events met study entry criteria and were included in statistical analyses. Subjects were excluded for undergoing initial surgery without confirmed pre-operative malignancy (n=393), pre-operative diagnosis of DCIS (n=936), pre-operative malignancy type unknown (n=16), known Stage IV breast cancer (n=6), confirmed breast cancer in an axillary node with no known primary (n=5), inflammatory breast cancer (n=75), pre-operatively known multifocal breast cancer (n=96), previous ipsilateral breast cancer (n=85), previous history of radiation therapy (n=98), and patients receiving endocrine-only neoadjuvant therapy (n=13). The remaining patients did not have identifiable clinical factors limiting eligibility for BCT. Of these patients, 111/2907 (3.8%) were treated with NAC. 

Patient demographics according to utilization of NAC are shown in Table 1. Younger women (< age 35) were more likely to receive NAC (*P*<0.0001). Additional pre-operative variables associated with higher utilization rates of NAC included known positive nodal status (*P*<0.0001), increasing tumor size (*P*<0.0001), negative estrogen receptor (ER) status (*P*<0.0001), and negative progesterone receptor (PR) status (*P*<0.0001). There was no statistically significant difference in NAC use between invasive ductal carcinoma and invasive lobular carcinoma (*P*=0.1041). Utilization of NAC varied significantly across the four treatment facilities (*P*<0.0001).

**Table 1:** Patient Demographics according to utilization of neoadjuvant chemotherapy.

	**Value**	**Initial surgery (N)**	**Neoadjuvant treatment (N)**	**Neoadjuvant treatment %**	**P-value**
Age	<35	22	5	22.73	
N=2907	35-44	240	21	8.75	
	45-54	670	50	7.46	<0.0001
	55-64	794	22	2.77	
	65-74	616	10	1.62	
	>75	565	3	0.53	
Ethnicity	African American	60	3	5.00	
N=2907	Asian	72	1	1.39	
	Hispanic	54	2	3.70	0.3087
	White/ Non-Hispanic	2287	95	4.15	
	Other/Unknown	434	10	2.30	
Institution	1	713	60	8.42	
N=2907	2	1017	11	1.08	<0.0001
	3	922	23	2.49	
	4	255	17	6.67	
Preoperative tumor size	0-10 mm	646	6	0.93	
N=2558	>10-20mm	1112	24	2.16	
	>20-30mm	487	21	4.31	<0.0001
	>30-40 mm	177	10	5.65	
	>40-50 mm	60	9	15.00	
	>50 mm	76	12	15.79	
Preoperative diagnosis	IDC	2439	101	4.14	
N=2907	ILC	282	7	2.48	0.1041
	Type Unknown	186	3	1.61	
ER status	Positive	2423	70	2.89	<0.0001
N=2887	Negative	464	41	8.84	
PR status	Positive	2223	62	2.79	<0.0001
N=2887	Negative	664	49	7.38	
Preoperative nodal status	Negative	2655	43	1.62	<0.0001
N=2907	Positive	252	68	26.98	
Initial surgery	Partial mastectomy	2202	60	2.72	<0.0001
N=2907	Total mastectomy	705	51	7.23	

Abbreviations: BCT, breast conservation therapy; ER, estrogen receptor; PR, progesterone receptor; IDC, invasive ductal carcinoma; ILC, invasive lobular carcinoma

A multivariable analysis of the patient, institution, and pre-operative tumor characteristics related to NAC use in the univariate analysis described above was performed (Table 2). Pre-operative positive nodal status, treating institution, pre-operative tumor size, and age were significantly associated with increased NAC utilization after controlling for other variables in the model (Table 2). Of note, ER and PR status were not independently significant.

**Table 2:** Multivariable analysis for association with neoadjuvant chemotherapy use.

	**Value**	**OR**	**95% CI**	**P-value**
Age	<35	6.77	1.33-34.46	0.021
	35-44	3.68	1.32-10.29	0.013
	45-54	3.92	1.62-9.5	0.003
	55-64	2.31	0.90-5.96	0.083
	65-74	ref		
	≥75	0.44	0.11-1.88	0.269
Institution	1	ref		
	2	0.08	0.03-0.22	<0.0001
	3	0.61	0.33-1.10	0.101
	4	1.31	0.63-2.75	0.469
Preoperative tumor size	0-10 mm	ref		
	>10-20 mm	1.29	0.50-3.35	0.595
	>20-30 mm	1.8	0.67-4.85	0.243
	>30-40 mm	2.24	0.72-6.97	0.164
	>40-50 mm	6.27	1.77-22.22	0.004
	>50 mm	5.69	1.75-18.44	0.004
ER status	Positive	0.81	0.36-1.84	0.612
	Negative	ref		
PR status	Positive	0.59	0.27-1.28	0.183
	Negative	ref		
Preoperative nodal status	Negative	ref		
	Positive	10.17	6.05-17.08	<0.0001
Initial surgery	BCT	ref		
	Mastectomy	1.17	0.66-2.08	0.589

Abbreviations: OR, odds ratio; CI, confidence interval; BCT, breast conservation therapy; ER, estrogen receptor; PR, progesterone receptor

**Figure 1:**
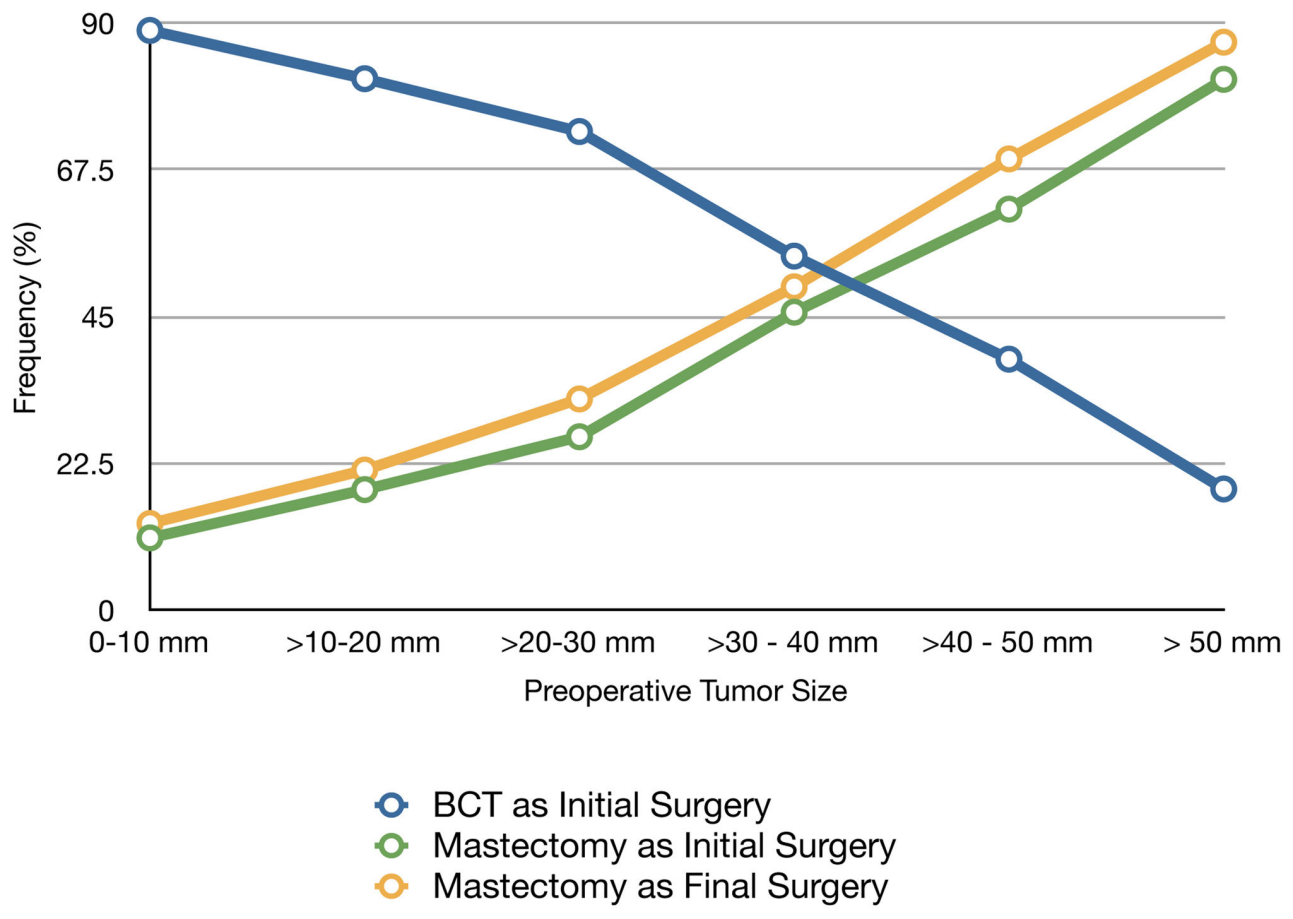
Frequency of Surgery Modalities according to Tumor Size As tumor size increases, the frequency of breast conservation therapy (BCT) decreases and the frequency of mastectomy as initial or final surgery increases. At a tumor size of >30 - 40 mm, an approximate 1:1 ratio exists between BCT and mastectomy.

The frequencies of PM and TM as the initial or final surgical procedures were examined (Figure 1). An estimated clinical tumor size of 30–40 mm was noted to have an approximately 1:1 ratio for frequency of initial PM to initial TM. BCT was used more often for smaller tumors, while initial TM and final TM were more common for larger tumors. When these data were examined according to individual institution, the trend for increased initial TM (exceeding 50% in tumors >40 mm) was seen at each institution and was statistically significant (*P*<0.0001) (Table 3). 

**Table 3:** Percent of initial mastectomy and neoadjuvant therapy according to tumor size by institution.

	**Institution 1**		**Institution 2**		**Institution 3**		**Institution 4**		**P value**	
Pre-operative tumor size	Initial mastectomy	NAC	Initial mastectomy	NAC	Initial mastectomy	NAC	Initial mastectomy	NAC	Initial mastectomy	NAC
0-10 mm	7.3	2.4	16.4	0.0	16.2	0.0	8.6	2.9	0.0199	0.0135
>10-20 mm	9.2	5.8	21.2	0.3	27.2	0.7	24.1	3.6	<0.0001	<0.0001
>20-30 mm	21.5	8.3	35.4	1.1	27.9	2.6	21.6	13.5	0.0477	0.0005
>30-40 mm	34.1	11.4	58.6	1.4	40.4	7.7	63.6	0.0	0.0318	0.1068
>40-50 mm	57.1	21.4	76.2	4.7	50.0	15.0	80.0	40.0	0.2952	0.2050
>50 mm	88.5	3.9	81.8	0.0	78.3	43.4	60.0	20.0	0.4900	<0.001
**P value**	<0.0001	0.022	<0.0001	0.045	<0.0001	<0.0001	<0.001	0.002		

Abbreviations: NAC: neoadjuvant chemotherapyOverall, initial surgery was PM for 60 (2.7%) patients and and TM for 51 (7.2%) patients who received NAC (*P*<0.0001). Of the 60 patients whose initial surgery was PM, achievement of BCT occurred in 53 patients (88.3%) and 7 (11.7%) required further surgery (*P*=0.0905). Utilization of NAC increased with clinical tumor size, yet less than 16% of tumors >50 mm in clinical size were treated with NAC (Table 1). Larger tumors were noted to have an increasing initial TM rate compared to smaller tumors (Figure 1), with the initial TM rate >80% for tumors with clinically estimated size >50 mm. Increasing utilization of NAC for increasing tumor size was also noted when NAC use was analyzed according to each individual institution (Table 3). Interestingly, however, three of the four institutions had lower rates of NAC for tumors >50 mm compared to tumors 40-50 mm at each respective institution. The frequency of BCT failure also increased with increasing clinical tumor size, reaching 29% in tumors >50 mm in size (Figure 2).

**Figure 2:**
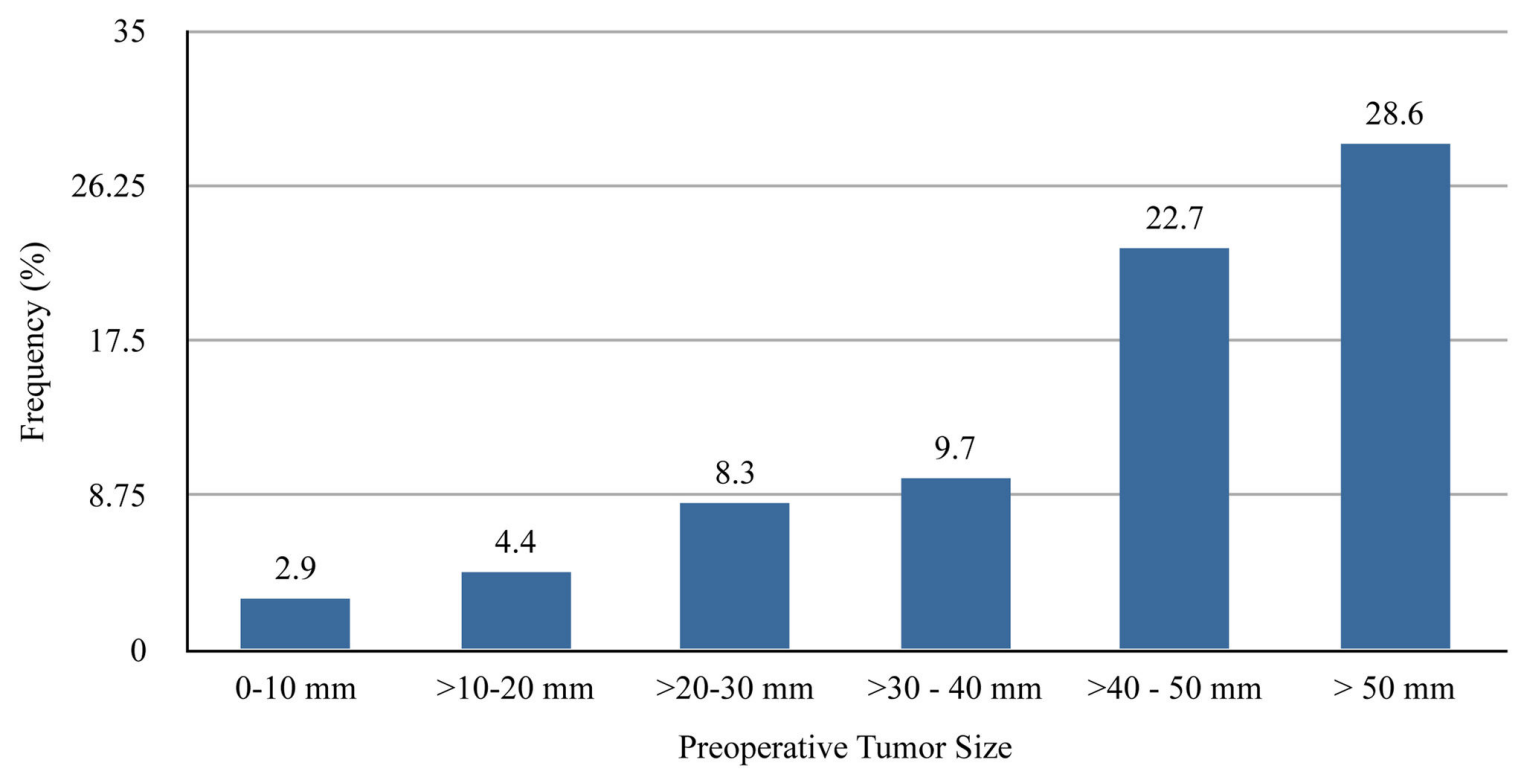
Frequency of BCT Failure by Tumor Size As tumor size increases, the rate of breast conservation therapy (BCT) failure increases. Frequencies are indicated above each bar.

## Discussion

Clinical trials have demonstrated the benefit of NAC for increasing breast conservation rates without compromising overall survival compared to adjuvant chemotherapy [16-18,24-28]. We found that NAC use varied significantly across four large institutions, suggesting non-uniform practice patterns among the participating institutions, and potentially throughout the country. The data presented here also suggest that NAC may be relatively underutilized as a means of tumor down-staging as evidenced by BCT failure. Interestingly, the percentage of patients undergoing TM as a first procedure increased dramatically for tumor sizes >3.0 cm, rather than the 5.0 cm size traditionally evaluated in clinical trials examining the effectiveness of NAC in tumor downstaging [1,18]. A similar finding was reported in a previous single institution study [29]. The present study demonstrates that a marked increase in initial TM for patients with tumors >3.0 cm exists across multiple institutions. Our findings suggest that in addition to larger tumor size, factors such as younger age and positive nodal status are also associated with increased NAC use. NAC was used infrequently in patients aged 65 or older. Lower use in the elderly may reflect higher co-morbidity or less patient concern with breast preservation. 

Common practice would suggest that NAC use would increase with increasing tumor size, as smaller tumors are more likely to be amenable to proceeding with BCT without requiring NAC. However, we did not observe a proportional increase in utilization of NAC with larger pre-operative tumor size, especially in tumors >4 cm in size (Table 1). When compared to the frequency of TM as the initial surgery, utilization of NAC is variable among the institutions, even for larger tumors. The rates of NAC use in tumors >5 cm were 3.9%, 0%, 43.5%, and 20% for the four institutions, despite frequencies of initial TM as the initial surgery of 88.5%, 81.8%, 78.3%, and 60%, respectively (Table 3). Data from these four institutions, all of which show a predilection for BCT in patients with smaller tumors, demonstrate potentially concerning variation in the utilization of NAC. At one institution, NAC use was extremely infrequent (1.1%). Two of the institutions had slightly more prevalent use (8.4% and 6.7%), but one of these surprisingly had lower utilization for tumor size >5 cm.

It is possible that many women who underwent TM as the initial procedure may have chosen TM as their treatment of choice, but we are unable to evaluate patient decision-making or education regarding potential NAC use in our study. Nevertheless, given that the majority of women with smaller breast tumors in this study chose initial BCT, it is not unreasonable to assume that women with larger tumors would also desire BCT if feasible. The large disparity between NAC use and BCT failure (compare Table 1 with Figure 2) suggests a potential opportunity to increase the rate of breast conservation through the greater use of NAC.

Variation in NAC use among various treatment centers deserves further investigation into the possible causes. Legitimate variation could result from inherent differences in the patient population or presentation of disease. However, we sought to minimize these differences by careful selection of study inclusion and exclusion criteria. Despite strict selection criteria, variation in NAC use persisted across institutions. There are several potential driving factors that may account for this finding including discrepancies in available resources such as multidisciplinary breast clinics, timing of multidisciplinary tumor conferences relative to time of initial surgery, level of training, and local culture [30].

A major limitation of the present study is the retrospective nature of data collection, which limited the determination of other treatment decision-making factors. Individual patient or surgeon preference was not available for review. Women with comorbidities precluding chemotherapy, strong family history or genetic risk factors such as BRCA status, or tumors with an extensive intraductal component may account for some of the omission of NAC [7]. Patient awareness of NAC as an option before surgery and evaluation of how it may impact the success of their initial surgical therapy could not be evaluated. Similarly, prospective involvement of medical oncologists or utilization of multidisciplinary tumor conferences before treatment initiation were not evaluated as part of this study. Gene expression profile testing was not widely implemented during the timeframe of this study, and therefore, should not have impacted the results. HER2-neu status was not available, as it was not consistently tested during the study time period. Type of NAC used was not available, and specific information regarding accessibility and enrollment in clinical trials was not available. Despite these limitations, the findings of the present study suggest further investigation may be useful for optimizing patient treatment preferences and initial treatment strategy. Strategies to reduce clinical variation and match best initial treatment selection with patient desired outcomes are essential steps in quality improvement.

Management of invasive breast cancer requires an effective multi-disciplinary program. Early involvement of medical oncologists in treatment discussions may impact the utilization of NAC for newly diagnosed patients who desire BCT. Institutional variation in the use of NAC may represent an opportunity to enhance quality in overall breast cancer care. Understanding the factors that influence this variation and potential underuse of NAC may advance efforts to improve treatment outcomes and optimize patient treatment choices and patient satisfaction in breast cancer care. Further investigation into improving both patient and clinician education is crucial.
